# A framework of biomarkers for visual system aging: a consensus statement by the Aging Biomarker Consortium

**DOI:** 10.1093/lifemedi/lnaf023

**Published:** 2025-06-28

**Authors:** Aging Biomarker Consortium, Chao Ma, Boxin Geng, Yangqing Zhang, Shan Li, Ruiyang Li, Wenben Chen, Si Wang, Weiqi Zhang, Jing Qu, Yun Feng, Qingfeng Liang, Kangxin Jin, Yonghao Gu, Wenru Su, Xuxiang Zhang, Wenjuan Zhuang, Jihong Wu, Zhaoyang Wang, Shengping Hou, Jiaxu Hong, Honghua Yu, Biao Yan, Mingguang He, Fan Lv, Guang-hui Liu, Gang Pei, Qingjiong Zhang, Tian Xue, Zi-Bing Jin

**Affiliations:** Beijing Tongren Hospital, Beijing Institute of Ophthalmology, Beijing Tongren Eye Center, Capital Medical University, Beijing Key Laboratory of Ophthalmology & Visual Science, Beijing 100005, China; Beijing Tongren Hospital, Beijing Institute of Ophthalmology, Beijing Tongren Eye Center, Capital Medical University, Beijing Key Laboratory of Ophthalmology & Visual Science, Beijing 100005, China; Beijing Tongren Hospital, Beijing Institute of Ophthalmology, Beijing Tongren Eye Center, Capital Medical University, Beijing Key Laboratory of Ophthalmology & Visual Science, Beijing 100005, China; School of Basic Medical Sciences, Capital Medical University, Beijing 100069, China; Beijing Tongren Hospital, Beijing Institute of Ophthalmology, Beijing Tongren Eye Center, Capital Medical University, Beijing Key Laboratory of Ophthalmology & Visual Science, Beijing 100005, China; State Key Laboratory of Ophthalmology, Zhongshan Ophthalmic Center, Sun Yat-sen University, Guangdong Provincial Key Laboratory of Ophthalmology and Vision Science, Guangdong Provincial Clinical Research Center for Ocular Diseases, Guangzhou 510060, China; State Key Laboratory of Ophthalmology, Zhongshan Ophthalmic Center, Sun Yat-sen University, Guangdong Provincial Key Laboratory of Ophthalmology and Vision Science, Guangdong Provincial Clinical Research Center for Ocular Diseases, Guangzhou 510060, China; Advanced Innovation Center for Human Brain Protection, National Clinical Research Center for Geriatric Disorders, Aging Translational Medicine Center, Beijing Municipal Geriatric Medical Research Center, Beijing Key Laboratory of Environment and Aging, Xuanwu Hospital Capital Medical University, Beijing 100053, China; China National Center for Bioinformation and Beijing Institute of Genomics, Chinese Academy of Sciences, Beijing 100101, China; Beijing Institute of Genomics, University of Chinese Academy of Sciences, Beijing 101408, China; State Key Laboratory of Organ Regeneration and Reconstruction, Institute of Zoology, Chinese Academy of Sciences, Beijing 100101, China; Beijing Institute for Stem Cell and Regenerative Medicine, Beijing 100101, China; Beijing Institute of Heart Lung and Blood Vessel Diseases, Beijing Anzhen Hospital, Capital Medical University, Beijing 100029, China; University of Chinese Academy of Sciences, Beijing 100049, China; Department of Ophthalmology, Peking University First Hospital, Beijing 100034, China; Beijing Tongren Hospital, Beijing Institute of Ophthalmology, Beijing Tongren Eye Center, Capital Medical University, Beijing Key Laboratory of Ophthalmology & Visual Science, Beijing 100005, China; Beijing Tongren Hospital, Beijing Institute of Ophthalmology, Beijing Tongren Eye Center, Capital Medical University, Beijing Key Laboratory of Ophthalmology & Visual Science, Beijing 100005, China; Department of Ophthalmology, First Affiliated Hospital of University of Science and Technology of China, Hefei 230001, China; Department of Ophthalmology, State Key Laboratory of Visual Science Shanghai Ninth People’s Hospital, Shanghai Jiao Tong University School of Medicine, Shanghai 200011, China; Department of Ophthalmology, Xuanwu Hospital, Capital Medical University, Beijing 100037, China; Department of Ophthalmology, People’s Hospital of Ningxia Hui Autonomous Region, Yinchuan 750011, China; Department of Opthalmology, Eye and ENT Hospital, Fudan University, Shanghai 200031, China; Shanghai Key Laboratory of Visual Impairment and Restoration, Science and Technology Commission of Shanghai Municipality, Shanghai 200031, China; Key Laboratory of Myopia and Related Eye Diseases, NHC, Shanghai 200031, China; Key Laboratory of Myopia and Related Eye Diseases, Chinese Academy of Medical Sciences, Shanghai 200031, China; Beijing Tongren Eye Center, Beijing Tongren Hospital, Capital Medical University, Beijing 100005, China; Beijing Tongren Hospital, Beijing Institute of Ophthalmology, Beijing Tongren Eye Center, Capital Medical University, Beijing Key Laboratory of Ophthalmology & Visual Science, Beijing 100005, China; Department of Ophthalmology, Eye & ENT Hospital, State Key Laboratory of Medical Neurobiology and MOE Frontiers Center for Brain Science, Fudan University, Shanghai 200031, China; Guangdong Eye Institute, Department of Ophthalmology, Guangdong Provincial People’s Hospital (Guangdong Academy of Medical Sciences), Southern Medical University, Guangzhou 510080, China; Department of Ophthalmology, Shanghai General Hospital, Shanghai Jiao Tong University School of Medicine, Shanghai 200080, China; The Hong Kong Polytechnic University, Hong Kong 999077, China; State Key Laboratory of Ophthalmology, Optometry and Visual Science, Eye Hospital, Wenzhou Medical University, Wenzhou Institute, University of Chinese Academy of Sciences, Wenzhou 325041, China; Wenzhou Institute, University of Chinese Academy of Sciences, Wenzhou 325001, China; State Key Laboratory of Organ Regeneration and Reconstruction, Institute of Zoology, Chinese Academy of Sciences, Beijing 100101, China; University of Chinese Academy of Sciences, Beijing 100049, China; Beijing Institute for Stem Cell and Regenerative Medicine, Beijing 100101, China; Collaborative Innovation Center for Brain Science, School of Life Science and Technology, Tongji University, Shanghai 200092, China; State Key Laboratory of Ophthalmology, Zhongshan Ophthalmic Center, Sun Yat-sen University, Guangdong Provincial Key Laboratory of Ophthalmology and Visual Science, Guangzhou 510060, China; Department of Ophthalmology, The First Affiliated Hospital of USTC, State Key Laboratory of Eye Health, School of Life Sciences, Division of Life Sciences and Medicine, University of Science and Technology of China, Hefei 230026, China; Beijing Tongren Hospital, Beijing Institute of Ophthalmology, Beijing Tongren Eye Center, Capital Medical University, Beijing Key Laboratory of Ophthalmology & Visual Science, Beijing 100005, China

## Abstract

The visual system is essential for human perception, converting light signals into electrical impulses and transmitting them to the brain to process environmental information. As individuals age, their physiological functions gradually decline, leading to age-related vision impairment that significantly impacts the quality of life in elderly individuals. China is home to the world’s largest aging population and faces significant challenges in combating visual system aging through effective treatments and interventions. In response to this challenge, the Aging Biomarker Consortium (ABC) of China has developed a consensus statement on biomarkers of visual system aging by integrating cutting-edge global research and synthesizing evidence-based medicine with clinical expertise. This consensus provides a multi-dimensional evaluation framework encompassing functional, morphological, and molecular biomarkers. Validated biomarkers for each domain are recommended not only to facilitate the early detection of vision changes but also to provide insights into the progression of age-related ocular diseases. By advancing this initiative, ABC aims to revolutionize visual health management in aging societies, ultimately improving outcomes for elderly populations in China and globally.

## Introduction

The visual system, composed of specialized physiological structures and neural mechanisms in organisms, serves as the fundamental apparatus for perceiving and processing external light signals. The visual system serves as a photoreceptive organ, transforming incoming photons into electrochemical neural signals. These signals traverse the retinogeniculate pathway to reach the occipital cortex, where they are synthesized into coherent visual perceptions. However, the visual system experiences a progressive decline in function with advancing age, which involves degenerative changes in multiple ocular structures. Photoreceptor cells and retinal pigment epithelial (RPE) cells in the macula, undergo functional decline, potentially contributing to the development of retinal diseases such as age-related macular degeneration (AMD). Moreover, the prevalence of AMD exceeds 10% among individuals aged ≥65. In addition, the progressive loss of retinal ganglion cells and weakened intraocular pressure regulation further elevate the risk of glaucoma [1]. The aging process of the visual system is associated with a number of factors, including cognitive decline, social impairment, and mental health issues. These conditions have a significant impact on the quality of life of elderly individuals [2, 3].

As the world’s most populous aging society, China faces unprecedented challenges posed by age-related diseases. Within the extant healthcare framework, there is an absence of biomarkers that are standardized and capable of accurately quantifying physiological age, structural integrity, and functional capacity of the visual system. Current clinical interventions for age-related visual system disorders are limited. In order to address this problem, the Aging Biomarker Consortium (ABC) of China organized an expert symposium in Beijing focusing on aging biomarkers in the visual system. The expert panel, through integrating cutting-edge global research and synthesizing evidence-based medicine with clinical expertise, developed a consensus statement on biomarkers of visual system aging. This consensus provides a multidimensional evaluation framework encompassing functional, morphological, and molecular biomarkers. Validated biomarkers for each domain are recommended to assess: (i) the current aging status, (ii) the rate of aging, and (iii) susceptibility to disease. The consensus statement also establishes a methodological foundation for future clinical investigations. By advancing this initiative, ABC aims to revolutionize visual health management in aging societies, ultimately improving outcomes for elderly populations in China and globally.

## Recommended methodology for biomarkers for visual system aging

The levels of recommendation and evidence in this consensus are presented in accordance with internationally recognized standards [4]. The details of the evidence levels and the strength of recommendations are provided in Table 1. All recommendations underwent rigorous review and discussion among ABC members, ensuring the integration of diverse perspectives and considerations into this consensus.

**Table 1.** Class of recommendations and level of evidence

Class (strength) of recommendation	Level (quality) of evidence
**Class I (strong) benefit >>> risk** **Suggested phrases for writing recommendation** • Recommended/indicated • Evidence and/or general agreement that a given treatment or procedure is beneficial, useful, and effective	**Level A** • Data derived from multiple randomized clinical trials or meta-analyses
**Class IIa (moderate) benefit >> risk** **Suggested phrases for writing recommendation** • Should be considered • Weight of evidence/opinion is in favor of usefulness/efficacy	**Level B** • Data derived from a single randomized clinical trial or large non-randomized studies
**Class IIb (weak) benefit ≥ risk** **Suggested phrases for writing recommendation** • May be considered • Usefulness/efficacy is less well established by evidence/opinion	**Level C** • Consensus of expert opinion, and/or small studies, retrospective studies, registries
**Class III (strong) risk > benefit** **Suggested phrases for writing recommendation** • Not recommended • Evidence/general agreement that the given treatment/procedure is not useful/effective and sometimes maybe harmful	**Note:** COR and LOE are determined independently (any COR may be paired with any LOE). COR, class of recommendation; LOE, level of evidence.

## Classification of visual system aging biomarkers

**Figure 1.**
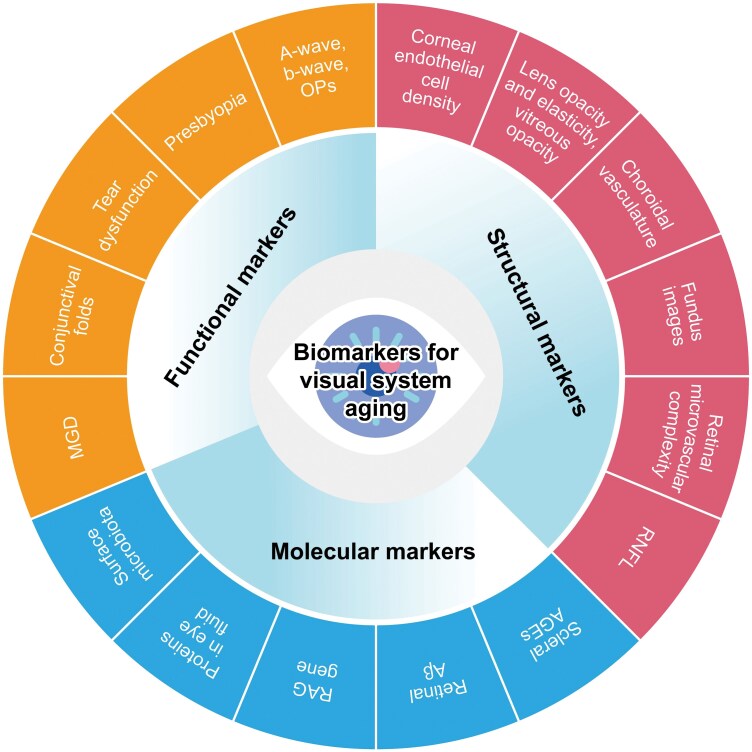
**Summary of proposed recommended markers of visual system aging.** The proposed assessment framework for visual system aging encompasses three dimensions: molecular, functional, and structural biomarkers. It comprehensively covers a broad spectrum of changes that take place at every level of the visual system throughout the aging process. These biomarkers hold great promise for extensive application in routine clinical practice. Nevertheless, it is crucial to stress that additional validation efforts are required to evaluate the efficacy of these biomarkers in the assessment of the biological aging of the visual system.

The aging of the visual system is a complex, multi-dimensional, and multi-layered biological process, involving molecular, cellular, organ, and systemic alterations. Biomarkers of visual system aging serve as critical biological indicators that effectively reflect the actual age, function, and structure. These biomarkers play a pivotal role in predicting the extent and rate of visual system aging, assessing the risk of visual system-related diseases, and evaluating the efficacy of anti-aging interventions. This consensus establishes a comprehensive evaluation framework that integrates functional, structural, and molecular biomarkers (Fig. 1).

### Functional markers of visual system aging

The principal clinical manifestations of visual system aging include diminished visual acuity, color vision abnormalities, and increased intraocular pressure. These manifestations are often closely associated with the development of various visual system diseases, particularly cataracts, glaucoma, and macular degeneration, with their prevalence rising with age [5, 6].

#### Degenerative changes in the eyelids

Aging is accompanied by a variety of degenerative changes occurring in the eyelid region, manifesting in conditions such as ptosis, entropion or ectropion, conjunctivochalasis, and meibomian gland dysfunction (MGD). Recent research has identified a decline in the proliferation signals of senescent meibomian stem cells, inadequate neural innervation of the meibomian glands, and a loss of type I collagen in niche fibroblasts [7]. A substantial correlation between MGD severity and aging has been demonstrated in both human and animal studies [8]. According to the *Chinese expert consensus on meibomian gland dysfunction: diagnosis and management (2023)*, the prevalence of meibomian gland dysfunction among individuals over 40 years old in China ranges from 54.7% to 68.3% [9]. Its assessment is based on multiple parameters, including meibomian gland orifice, meibum quality, and meibomian gland expressibility. Artificial intelligence (AI) can be utilized to analyze the morphological parameters of meibography in the elderly, which enables a comprehensive evaluation of meibomian gland status [10].

An increase in conjunctival folds is yet another age-related degenerative phenomenon. As age advances, the elderly frequently exhibit varying degrees of conjunctival laxity, typically characterized as subconjunctival tissue relaxation and folding. A Japanese epidemiological study revealed that the prevalence of conjunctivochalasis exceeded 98.0% in individuals > 61 years old [11]. Meanwhile, a Shanghai-based study in China found that conjunctivochalasis was present in 44.08% of individuals > 60 years old [12]. The severity of conjunctivochalasis is strongly age-dependent, as observed via slit lamp microscopy and anterior segment optical coherence tomography (OCT) [13]. Clinically, the Lid-parallel conjunctival folds (Lipcof) grading system is often utilized to quantify the severity of conjunctival folds [14].

In summary, MGD and increased conjunctival folds can be regarded as functional markers of visual system aging. However, it is essential to exclude non-age-related pathogenic factors.

#### Tear dysfunction

As individuals age, the density of corneal nerves gradually decreases, particularly within the corneal epithelial and stromal layers. This decline has been demonstrated to contribute to tear dysfunction in the elderly, which may also be closely linked to diminished lacrimal and meibomian gland function. Epidemiological studies indicate that the prevalence of dry eye is about 20% among individuals aged 30–40 years old, increasing to 36.1% in those > 70 years old [15]. As described in the *Chinese expert consensus on dry eye: definition and classification (2024)*, slit lamp examination, and tear film break-up time testing are essential approaches for evaluating dry eye symptoms (Table 2). Consequently, tear dysfunction may serve as a potential indicator of visual system aging. A comprehensive assessment of aging should incorporate other markers of visual system degeneration to provide a more holistic evaluation.

**Table 2.** Grades of dry eye by Chinese Medical Association (CMA) in 2024

Classification	Slit lamp examination	Tear film break-up time (TBUT)
Mild	No significant ocular surface damage (corneal fluorescein staining spots < 5)	5 s < TBUT ≤ 10 s
Moderate	Corneal damage limited to no more than 2 quadrants and/or corneal fluorescein staining spots ≥ 5 but < 30	2 s ≤ TBUT ≤ 5 s
Severe	Corneal damage involving 2 or more quadrants and/or corneal fluorescein staining spots ≥ 30, with fluorescein staining spot fusion forming coarse spots, plaques, or filamentous structures	TBUT < 2 s

#### Abnormal visual function

##### Presbyopia

The accommodation function of the ciliary muscle, along with the stability of the lens in terms of shape and rigidity, plays a vital role in maintaining clear near vision. However, as aging progresses, the regulatory function of the ciliary muscle gradually declines, primarily manifesting in decreased muscle elasticity, reduction in amplitude of accommodation, and slowed pupillary response. In addition, the progressive loss of lens elasticity exacerbates the decline in ocular accommodation.

Globally, about 1.8 billion people are affected by presbyopia, and the number is still on the rise [16]. Presbyopia typically begins around the age of 45 and gradually worsens with aging. Most emmetropic individuals require an additional refractive power of approximately +1.50 D at age 45, around +2.00 D by age 50, and approximately +3.00 D by age ≥ 60. The progression is strongly correlated with aging, indicating it is a physiological phenomenon that develops gradually alongside the natural aging process.

The function of the ciliary muscle is evaluated by measuring the eye’s accommodative ability from the far point to the near point. Negative relative accommodation and positive relative accommodation can, respectively, test the relaxation and contraction of the ciliary muscle. Meanwhile, the RAF near-point rule not only detects the decline in the lens’s amplitude of accommodation but also monitors the progressive recession of the near point of accommodation [17].

##### Retinal functional degeneration

The macular region, the most critical area of the eye responsible for fine central vision, is susceptible to degenerative changes over time, leading to functional loss in the central retina. Clinically, this often manifests as blurred vision or central scotoma. A comprehensive evaluation of visual function includes perimetry to assess central and peripheral visual field defects, along with tests for visual acuity, contrast sensitivity, and color vision to ensure accurate disease diagnosis.

At the cellular level, age-related retinal changes can be detected using electroretinography (ERG), which reveals a progressive reduction in scotopic a-wave and photopic b-wave amplitudes, directly reflecting functional decline in rod and cone photoreceptors [18]. Notably, oscillatory potentials (OPs), which serve as key indicators of inner retinal integrity, undergo changes that precede alterations in a-waves and b-waves, exhibiting greater sensitivity to age-related changes, particularly within low-frequency bands. Furthermore, Morlet wavelet transform, an advanced signal analysis technique, complements conventional time–amplitude analysis. This renders it a highly sensitive tool for the assessment of age-related retinal functional decline [19].

##### Recommendations:

1. MGD may serve as a functional biomarker for visual system aging, with clinical evaluation possible through slit lamp examination and meibography. It is essential to differentiate physiological aging from other potential etiological factors during diagnosis (Level A, Class ІІ a).
2. Conjunctival folds may serve as a functional biomarker for predicting visual system aging, with their increase potentially indicating visual system aging. They can be assessed through slit lamp microscopy clinically (Level A, Class ІІ a).
3. Tear dysfunction can reflect the degree of visual system aging, clinically evaluable through tear secretion tests. It is recommended to use with other visual system aging biomarkers (Level A, Class ІІ a).
4. Presbyopia may serve as a functional biomarker for visual system aging, which can be analyzed through visual acuity testing (Level A, Class І).
5. The decreased amplitudes of ERG parameters (a-wave, b-wave, and OPs) suggest a functional decline in cells during visual system aging (Level A, Class І).

### Structural markers of visual system aging

The aging of the visual system is not merely manifested by a decline in its function but also by the aging of its tissue structure. These structural alterations often serve as the foundation for the onset and progression of visual system diseases. In consequence, the understanding of the structural characteristics of visual system aging is of great significance for the prediction of disease risk.

#### Decrease in the number of corneal endothelial cells

As the body ages, a series of degenerative changes occur in the structure and function of the cornea. The principal features comprise the thickening of the corneal epithelium and the endothelial basement membrane, particularly Descemet’s membrane of the endothelial basement membrane [20]. Moreover, the density of nerves within the corneal epithelial plexus diminishes with aging. Both the reduction in the number of corneal endothelial cells and the presence of their morphological abnormalities are significant features of corneal senescence in the elderly [21]. The density of endothelial cells naturally declines, decreasing from about 3000–3500 cells/mm² in young adulthood to around 2000 cells/mm² by the age of 60–70. In addition, the presence of crescent- or ring-shaped gray-white opacities at the corneal limbus is commonly observed in older individuals, which is known as “arcus senilis,” a typical manifestation of corneal aging. Changes in corneal endothelial cell density represent a key structural marker of visual system aging and can be assessed using *in vivo* confocal microscopy (IVCM) and optical coherence tomography (OCT).

#### Lens changes

Age-related cataracts (ARC) are a progressive degenerative condition characterized by lens opacification and color changes associated with aging. This pathological process typically emerges in individuals over the age of 50, and its incidence gradually rises.

The clinical manifestations of ARC primarily include lens opacity, pigment deposition, and reduced transparency, which may ultimately lead to visual impairment. To effectively assess the severity and extent of ARC, the “Lens Opacity Classification System II/III” (LOCS II/III) and the “Emery–Little cataract grading system” are widely employed in clinical practice [22, 23]. The LOCS II grading system categorizes cataracts based on the degree and location of lens opacities, while the Emery–Little classification evaluates lens nucleus color changes and hardness characteristics for classification. In addition, subjective scoring systems and anterior segment imaging analysis systems are used to provide a comprehensive diagnosis.

#### Vitreous liquefaction

Studies indicate that about 20% of the vitreous cavity experiences liquefaction between the ages of 14 and 18, and > 50% of the vitreous becomes liquefied by the age of 80–90 [24, 25]. This age-related vitreous syneresis may contribute to posterior vitreous detachment, which is most commonly observed in middle-aged and elderly populations (45–65 years). Severe liquefaction might increase the risk of retinal breaks and tractional retinal detachment. Importantly, the progressive accumulation of vitreous opacities often leads to visual obscurations.

#### Choroidal vascular changes

Age-related alterations occur in human choroidal vasculature, exhibiting a progressive reduction in overall choroidal thickness, vascular diameter, and choroidal blood flow. Fundoscopic examination typically reveals significant darkening of the red reflex. Concurrently, there is a marked decrease in the vascular density of choroidal capillaries, which is also linked with upregulated expression of chemokine and complement genes, elevated macrophage infiltration, and alterations in macrophage polarization [26]. These choroidal vascular changes reliably track visual system aging, providing validated structural biomarkers for senescence assessment.

#### Retinal changes in aging

##### Changes in tissue structure

Structural alteration in the retina, including a reduction in the number of specific neuronal groups, such as the inner nuclear layer, inner plexiform layer (INL), outer plexiform layer, and outer nuclear layer (ONL), have been observed. This phenomenon is accompanied by functional degeneration and has emerged as a key indicator in the study of degenerative retinal lesions. As the aging process advances, the retina undergoes a gradual thinning, with particularly pronounced atrophy in the ONL and a simultaneous decline in the number of retinal ganglion cells (RGCs). Electron microscopy observations of aging RGCs reveal an increase in axonal volume, an enlarged mean diameter of mitochondria within axons, and a reduced mitochondrial density, which renders RGCs more susceptible to degeneration and loss. Photoreceptors, one of the most vulnerable neuronal types, exhibit a significant reduction in number in aged mice [27]. In addition, other studies have reported similar findings and further identified a substantial thinning of the INL [28].

##### Changes in fundus image

Previous studies have employed analysis methods based on fundus image characteristics to assess age-related fundus changes, thereby elucidating several features of the aging fundus. For example, OCT reveals significant thinning of the retinal nerve fiber layer (RNFL). Semi-automatic retinal analysis software and confocal microscopy detect a narrowing of the diameters of arterial and venous blood vessels, along with an increase in vessel wall thickness. Collectively, these characteristics signify the progressive degeneration of retinal structure and function with aging [17].

A large-scale study involving samples from 11,052 healthy participants successfully developed a deep-learning model based on fundus images. By collecting 19,200 45-degree non-mydriatic and non-stereo fundus images centered on the macula, this model accurately predicts retinal age. The research team introduced the innovative concept of “retinal age gap (RAG),” defined as the difference between the retinal age predicted by the model and the chronological age, as a reliable measure of retinal aging. In this study, the absolute value of RAG was constrained to a range of 3.55 years [29]. Furthermore, the study demonstrated that retinal age can serve as a window to assess brain cell aging. An analysis of 35,834 participants without a history of Parkinson’s disease (PD) revealed that for every one-year increase in RAG, the risk of developing PD rises by 10% [30].

Interestingly, retinal microvascular complexity is expected to be a novel and highly promising biomarker of biological age. By using a hand-held, non-mydriatic fundus camera, researchers discovered that retinal microvascular complexity declines significantly with aging. In individuals with aging-related diseases, this decline occurs more than twofold faster than in those experiencing physiological aging. This important finding suggests that early identification of individuals with accelerated aging could enable timely intervention, thereby effectively postponing the onset of aging-related diseases [31].

##### Morphological changes of the optic nerve

Alterations in optic nerve fibers resemble age-related changes observed in nerve fibers throughout other regions of the central nervous system. These changes are primarily characterized by notable modifications in the myelin structure, with the thickening of the myelin cytoplasm emerging as one of the most prominent features. And it proceeds until the myelin eventually disintegrates. Furthermore, age-related changes in optic nerve fibers include a significant reduction in myelin-associated glycoproteins, the formation of myelin vesicles, and the phosphorylation of myelin. These structural modifications may impact the speed at which optic nerve fibers transmit visual information [32–35].

In mammals, optic nerve aging is chiefly characterized by distinct changes in the axons of RGCs and a gradual thinning of the RNFL around the optic papilla [36]. This transformation can be detected using spectral-domain optical coherence tomography (SD-OCT). While morphological changes in the optic nerve are considered a potential indicator for assessing visual system aging, current research remains largely confined to animal models, necessitating further clinical studies to validate its practical applicability.

##### Irregular morphology of RPE cells

Among individuals < 51 years of age, the majority of RPE cells in the foveal region maintain a polygonal shape and a consistent morphology, with 59% exhibiting a hexagonal structure. In the elderly > 80 years old, the proportion of hexagonal cells in the fovea decreases to 52%. The arrangement of RPE cells changes, and their morphology becomes irregular, yet the overall cell number remains stable [37]. Irregular RPE cell morphology may mirror retinal aging; however, due to the lack of efficient and non-invasive clinical testing methods, its use is not recommended at present.

##### Recommendations:

1. Corneal endothelial cell density may be considered for use as a structural biomarker for visual system aging, with reduced density suggesting potential aging. Clinical assessment can be performed via IVCM and OCT (Level B, Class I).
2. Lens opacity and elasticity, and vitreous opacity may be considered for use as structural biomarkers for visual system aging. Clinical evaluation can be conducted using slit lamp microscopy and fundoscopy (Level A, Class I).
3. Choroidal vasculature can be used as a structural biomarker for visual system aging. Clinical assessment is likely to be performed via fundus imaging (Level B, Class IIa).
4. RAG-based fundus images may be used as a structural biomarker for visual system aging. Clinical examination can be conducted using fundus imaging (Level B, Class IIa).
5. Retinal microvascular complexity can be implied as a structural biomarker for visual system aging, with diminished complexity indicating a potential aging process. Clinical assessment can be performed via a hand-held, non-mydriatic fundus camera (Level B, Class ІІ a).
6. RNFL can be a structural biomarker for visual system aging, with its thinning indicating potential aging. Clinical evaluation can be conducted via SD-OCT (Level C, Class ІІ a).

### Molecular markers of visual system aging

#### Scleral molecular markers

Advanced glycation end-products (AGEs) are compounds formed via non-enzymatic glycation reactions between sugars/metabolites and biological macromolecules. Their accumulation is commonly observed with aging, diabetes, oxidative stress, and chronic inflammation, causing widespread tissue damage. AGEs levels in corneal collagen and other ocular tissues (e.g. lens and Bruch’s membrane) show significant age-dependent elevation [38]. Non-invasive research has highlighted the age-related accumulation of AGEs in the sclera, although this progress occurs slower than Bruch’s membrane deposition [39]. The sclera represents a promising target for *in vivo* AGEs analysis in aging populations, with AGEs serving as potential molecular biomarkers for visual system senescence.

#### Corneal molecular markers

Age-related changes in corneal epithelium manifest as significantly increased permeability and gradual impairment of barrier function, primarily due to altered distribution of integrin subunits, particularly the discontinuous expression patterns of α6 and β4 subunits in hemidesmosomes [40].

In elderly individuals, there is a sharp decline in the DNA repair capability of corneal endothelial cells, accompanied by an increase in the level of the oxidative stress marker 8-hydroxy-2’-deoxyguanosine (8-OHdG), indicating cumulative oxidative stress in the eye. These effects accelerate corneal aging, leading to a reduction in corneal endothelial cell density and thinning of the corneal stroma [41]. Mouse models with DNA repair deficiencies demonstrate structural similarities to aged human corneas. Mitochondrial DNA (mtDNA) exhibits particular vulnerability to mutations. UV-A-induced damage triggers oxidative stress, and leads to mtDNA damage and characteristic mutations (e.g. the mtDNA T414G mutation), and ultimately results in corneal opacification and rigidity [42, 43].

The stem cell marker p63 shows age-dependent differences in regional distribution in rat corneas:

● Juvenile: Central (+) / Peripheral (−)
● Aged: Central (−) / Peripheral (+) [44]

The aforementioned non-specific markers currently lack effective and safe clinical testing methods, so they are not recommended temporarily.

#### Lens molecular markers

α-Crystallins, as the predominant structural proteins in the lens, belong to the small heat shock protein family and act as molecular chaperones in maintaining lens transparency. Significant structural alterations of α-crystallins are revealed to occur between the ages of 40 and 50, characterized by the reduction in soluble free-form proteins and the loss of high-molecular-weight proteins, ultimately promoting the formation of protein aggregates [45]. As a key contributor in cataract formation, the aggregates extensively bind to the lens fiber cell membrane, obstruct membrane pore structure, and hinder the transport of antioxidants. Concurrently, the lens gradually becomes yellow as a result of aging, principally caused by the accumulation of glutathione-3-hydroxykynurenine glucoside. This fluorophore accumulates in the lens nucleus and conducts cross-linking reactions with lens proteins. It is worth noting that the buildup of these aggregates increases light scattering, further exacerbating the loss of lens transparency [46]. Due to the lack of clinically feasible non-invasive detection methods, the use of lens molecular biomarkers is not advised.

#### Vitreous molecular markers

The vitreous body endures a process of progressive liquefaction, which is related to various ocular diseases, including posterior vitreous detachment, retinal tear, retinal detachment, etc. OCT reveals characteristic morphological alterations during vitreous aging:

● Thinning of the vitreous cortex in the fovea
● Progressive thickening of the vitreous cortex in the parafoveal area [47]

As an extracellular matrix with sparse cellular components, the vitreous is primarily composed of collagen and hyaluronic acid. Key molecular insights cover:

● A significant reduction in type II collagen and its degradation products [48]
● Elevation of plasminogen levels engaged in connective tissue degradation and remodeling
● Stable activity of matrix metalloproteinases (MMP-2 and MMP-9) [49]

The interaction mechanisms between these molecules require further investigation to elucidate their precise roles in vitreous aging and related pathologies.

#### Retinal molecular markers

##### Protein markers

β-Amyloid (Aβ), a core component of drusen, functions as a precise biological indicator for retinal aging. Emerging evidence reveals its dual association with AMD and neurodegenerative disorders like Alzheimer’s disease (AD) [50, 51]. Experimental findings demonstrate that exogenous Aβ elicits characteristic senescent phenotypes in RPE cells [52]. A noteworthy clinical study (*n* = 82 participants, 141 eyes) achieved > 90% specificity for preclinical AD diagnosis by combining plasma biomarkers (Aβ42/40 ratio, p-tau181, and p-tau217) and retinal Aβ-positron emission tomography (PET) imaging [53]. While Aβ deposition holds the potential for assessing visual system aging, a comprehensive differential diagnosis necessitates integrating fundus imaging features with systemic metabolic markers.

CD47, a transmembrane protein that forms a “don’t eat me” signal via SIRPα binding, also shows age-related decline in humans and mice. In the context of AMD, the downregulation of CD47 may facilitate the formation of melanosome-phagocytosing cells and propel the degeneration of RPE cells [54].

The aging RPE cells represent a central pathological element in AMD. Predominantly expressed in the RPE cells, bone morphogenetic protein 4 (BMP4) mediates oxidative stress-induced senescence in RPE cells via Smad and TAK1-p38 pathways [55]. In addition, apoptosis-related protein 3 (Apr3) has been implicated in oxidative stress and aging, with its expression being induced during replicative and premature senescence in RPE cells. Furthermore, its overexpression in ARPE-19 cells accelerates cellular aging [56]. Thus, further studies are necessary to provide a theoretical foundation for the specific expression patterns of BMP4 and Apr3 in physiological aging.

##### Genetic markers

###### Genome-wide association study (GWAS)

RAG serves not only as a valid indicator of retinal tissue aging but also as a systemic biomarker of biological aging, highlighting its potential as a comprehensive aging assessment metric. A landmark study integrated data from two large population cohorts—the UK Biobank and the Genetics of Diabetes Audit and Research in Tayside Scotland (GoDARTS), utilizing multi-trait GWAS analysis (MTAG). This study identified 13 independent single nucleotide polymorphisms significantly associated with RAG, particularly mutations in SH3YL1 and OCA2, which may contribute to the progression of senescence [57]. This discovery provides robust genetic evidence supporting RAG as a molecular biomarker for visual system aging.

###### Single-cell transcriptomics

Through comprehensive analysis of more than 119,520 single cells using single-cell RNA-sequencing (scRNA-seq) and assay for transposase-accessible chromatin with high-throughput sequencing (ATAC-seq) technologies, researchers have constructed detailed single-cell atlases of aged human and primate retinas [58]. The research includes:

● Identification of 11 major retinal cell types:
  ♦ 6 neuronal populations (rod, cone, horizontal, amacrines bipolar, and ganglion cells)
  ♦ 3 glial subtypes (Müller cells, astrocytes, and microglia)

Key findings are:

● Transcriptional dynamics:
  ♦ 87 upregulated genes, associated with hypoxic response, cell death regulation, and microglial activation
  ♦ 121 downregulated genes, involved in visual perception phototransduction, ATP biosynthesis, and retinol metabolism
● Significant cellular alterations:
  ♦ Depletion of MYO9A- rods and H2 horizontal cells
  ♦ Expansion of microglial populations
● Compared to the peripheral retina, regional variability in the foveal region demonstrates:
  ♦ Higher senescence scores
  ♦ Greater susceptibility to aging

These findings provide revolutionary resolution of cell type-specific aging patterns and reveal potential therapeutic targets for age-related retinal degeneration.

##### Epigenetic markers

###### CpG island methylation in regulatory regions

The elongase of very long-chain fatty acids like-2 (ELOVL2) gene encodes an enzyme involved in the synthesis of long-chain polyunsaturated fatty acids and is expressed across multiple tissues. The methylation of the ELOVL2 gene regulatory region has been identified as strong evidence of senescence in serum, establishing it as a promising epigenetic biomarker [59]. With advancing age, age-dependent hypermethylation of CpG islands is observed in the ELOVL2 promoter, accompanied by a gradual decline in its protein expression. Intravitreal injection of the demethylating agent 5-Aza-dc can reverse the hypermethylation of the ELOVL2 promoter, and thereby restore its expression levels [60]. Although methylation patterns of the ELOVL2 promoter in body fluids effectively reflect systemic aging trends, they lack tissue specificity. Consequently, this biomarker is not currently recommended for clinical use.

###### Histone

Histones are fundamental structural components of chromatin, playing irreplaceable roles in both gene expression regulation and chromatin structure maintenance. Histone depletion has been regarded as a distinctive hallmark of RPE aging in both human and animal models. In aged RPE, the researchers discovered:

● Reduction in core histone expression (H1, H2A, H2B, H3, and H4), which is consistent across ganglion cell layer (GCL), INL, and ONL
● Significant downregulation of histone-regulatory genes detected by transcriptomic profiling, especially histone locus body (HLB), including
  ♦ Histone nuclear factor P (Hinfp)
  ♦ Nuclear protein ataxia-telangiectasia (Npat)
  ♦ Caspase 8-associated protein 2 (Casp8ap2) [61]

Existing studies have also illuminated that dysregulation of histone modifications is bonded with the onset of neurodegenerative diseases (e.g. AD and PD). Hence, epigenetic research on histones holds vital importance in elucidating the mechanisms of cellular aging.

##### miRNA-related markers

microRNAs (miRNAs) represent a class of highly conserved endogenous small non-coding RNA molecules (typically 19–25 nucleotides in length) in eukaryotic cells. These molecules are essential in regulating various biological processes, including cellular proliferation, differentiation, metabolic homeostasis, developmental control, and disease pathogenesis. Notably, many miRNAs have been implicated in age-related diseases in vision systems, as they influence diverse biological processes [62].

###### let-7 family

The let-7 miRNA family are highly conserved miRNAs and is widely expressed in ocular tissues. Studies indicate that the let-7 family is involved in neurodegenerative disorders, with let-7d particularly contributing to disease progression through its regulation of neuroinflammatory responses and apoptotic pathways [63]. The expression levels of let-7d, along with let-7b and let-7c, are escalated in senescent retina and vitreous, respectively. Studies have also demonstrated that elevated let-7 levels enhance post-transcriptional hyaluronic acid production in Müller glial cells, thereby affecting the vision system and its related diseases.

###### miR-33

miR-33 mediates cholesterol efflux pathways in RPE, where its progressive age-dependent elevation contributes to dysregulated lipid homeostasis and leads to AMD development. ATP-binding cassette protein A1 (ABCA1), a membrane transport protein in RPE cells, is also responsible for maintaining lipid balance in the cell membrane. Of particular note, miR-33 directly targets the 3’ untranslated region (3’ UTR) of ABCA1, suppressing its expression. Age-related upregulation of miR-33 suppresses ABCA1 expression, which subsequently impairs cholesterol export and exacerbates lipid accumulation in RPE cells [64]. Targeted inhibition of miR-33 may attenuate pathological cholesterol deposition and reduce immune cell infiltration in the RPE layer, potentially mitigating the progression of AMD.

###### miR-34a

miR-34a has been established as a senescence biomarker through its upregulated expression in both the circulatory and neural systems. In the mouse brain, the upregulation of miR-34a is associated with neurodegenerative pathology and is involved in the p53 and Sirt1 signaling network. As an extension of the central neural system (CNS), the retina also exhibits comparable miR-34a dynamics with a steady increase in peaks at 24 months [64]. Further investigation is required to determine the translational relevance of these findings to human retinal aging.

###### miR-146 family

The miR-146 family, consisting of miR-146a and miR-146b, constitutes a pair of miRNAs with homologous sequences that play pivotal roles in retinal and choroidal aging processes. miR-146a increases in fibroblasts, umbilical vein endothelial cells, and trabecular meshwork cells, whereas both miR-146a and miR-146b levels rise in RPE cells, spanning from 2 to 48 months. Further insights indicate that overexpression of miR-146a mimics suppresses the expression of inflammatory factors IL-6 and VEGF-A in RPE cells, suggesting that miR-146a may regulate inflammatory responses during aging through a negative feedback effect [65].

###### Sex-specific miRNA markers

Emerging evidence reveals sexual dimorphism in miRNA expression profiles, which may differentially influence retinal structure and function as the body ages.

Sex-dependent expression patterns in aged mice include:

● Male-specific upregulation:
  ♦ miR-27a-3p
  ♦ miR-27b-3p
  ♦ miR-20a-5p
● Non-gender-specific downregulation:
  ♦ miR-20b-5p [66]

These sex-biased miRNA alterations may contribute to differential susceptibility to retinal disorders with aging.

##### Coenzyme markers

Coenzymes are a group of small, non-protein organic molecules that reversibly interact with enzymes to facilitate the catalysis of biochemical reactions. As people age, significant alterations in coenzyme levels and functionality influence the senescence process, including riboflavin (vitamin B2), coenzyme Q10 (CoQ10), and nicotinamide adenine dinucleotide (NAD^+^).

Upon conversion, riboflavin gives rise to two essential coenzymes: flavin adenine dinucleotide (FAD) and flavin mononucleotide (FMN), both of which are fundamental in cellular energy metabolism. As one of the most metabolically active tissues, the retina demands substantial energy to sustain visual function, necessitating high levels of FAD and FMN consistently. However, with increasing age, there is a progressive decline in FAD and FMN concentrations in the RPE and retina, a shift that may be linked to impairments in energy metabolism [18].

Around 40% loss in CoQ10 concentration is reported in aged retinas, accompanied by impaired antioxidant capacity and reduced ATP synthesis efficiency [67]. The supplement of CoQ10 may partially ameliorate age-related impairments. However, the *in vivo* detection of CoQ10 remains technically challenging, posing a barrier to its clinical application.

NAD^+^ participates in retina mitochondrial oxidative phosphorylation and fosters ATP production to meet the high-energy demands under light exposure. There is a decline in NAD^+^ levels with aging, a process parallel to the reduced expression of nicotinamide phosphoribosyltransferase (NAMPT) [68]. Therapeutic approaches targeting NAMPT and the NAD^+^ biosynthesis pathway have shown promise in mitigating RPE aging, further underscoring the critical role of NAD^+^.

##### Phospholipid markers

An integrative analysis of metabolic intermediates across multiple pathways has revealed that glycerophospholipid metabolites, such as phosphatidylcholine, possess unique advantages in sensitivity, specificity, and accuracy for predicting retinal aging [69]. Despite their potential as novel biomarkers, invasive detection methods have yet to become available.

#### Lacrimal gland markers

In aged mice, histopathological analysis shows a marked reduction in epithelial cells surrounding the ducts and mononuclear cell infiltration occupying 11.0% ± 6.5% of the glandular area. The ultrastructural examination further reveals periductal fibrosis, abnormal lipofuscin accumulation in the acinar cell cytoplasm, and mitochondrial damage characterized by swelling and disrupted cristae. At the molecular level, periductal monocytes and vascular endothelial cells show upregulated oxidative damage markers (8-OHdG, 4-HNE, and HEL) and enhanced expression of senescence-associated proteins such as p38 and p16 [70]. Immunological studies demonstrate elevated levels of MHC-II-expressing B cells in both the lacrimal glands and lymph nodes [71]. Lacrimal gland function recession is accompanied by reduced acetylcholine release and protein secretion. Meanwhile, the density and distribution of both parasympathetic and sympathetic nerves diminish with aging [72]. These findings provide important insights into the molecular mechanisms underlying lacrimal gland aging.

#### Fluid markers

Molecular markers in ocular fluids, including tears, aqueous humor, and vitreous humor, provide valuable insights for evaluating visual system biological terms due to their relative accessibility.

A study enrolled volunteers spanning an age range of 19–93 years (*n* = 75). Detected age-dependent elevations include:

● Inflammatory mediators (IL-8, IL-6, and RANTES)
● Tissue remodeling factors (MMP-1)
● 9 notable protein markers were assessed by protein microarray analysis [73]

However, given the relatively limited range of candidate proteins, future investigations should broaden to include other biomarker candidates, aiming for a more comprehensive understanding of the variations in tear composition throughout aging.

Another study utilizing 120 liquid biopsy samples from the aqueous and vitreous humor developed an unprecedented method named Tracing Expression of Multiple Protein Origins (TEMPO), which enables the discovery of the cellular origins within ocular fluids and promotes precise age prediction of the visual system. By integrating proteomics, single-cell transcriptomics, and AI algorithms, the study yielded several significant findings:

● Characterization of 6313 aqueous proteins with nonlinear aging patterns
● Identification of 26 core aging-related protein markers
● AI-driven elucidation of key cellular pathways in ocular aging [74]

To sum up, the integration of multi-omics data from ocular fluid facilitates minimally invasive monitoring and testing. However, extensive validation is necessary to ensure the reliability of these findings, which will require the implementation of large-scale cohort studies.

#### Microbiota markers

Variations occurred in microbial composition across meibum, conjunctival sac, and eyelid skin. A cross-sectional study (*n* = 36) employing 16S rRNA gene sequencing revealed distinct microbial signatures:

Elderly cohort (60–70 years):

● Lower diversity, dominated by *Corynebacterium* sp. and *Neisseriaceae*
● Greater resemblance between conjunctival sac and eyelid skin

Young cohort (20–35 years):

● Higher diversity in meibum and conjunctival sac, dominated by *Propionibacterium acnes*
● Greater resemblance between meibum and conjunctival sac

These findings suggest that ocular surface microbiota may serve as practical biomarkers for visual system aging, and can be detected via high-throughput sequencing following non-invasive swab sampling [75]. Given the limited sample size of this research, further clinical research is required to evaluate its practical efficacy.

#### Recommendations:

1. Scleral AGEs can potentially be considered as molecular biomarkers for predicting visual system aging, and an elevation in them can suggest the possibility of visual system aging. They can be examined by Raman spectroscopy (Level C, Class IIb).
2. Retinal Aβ deposition can act as a molecular biomarker for predicting visual system aging. Clinically, Aβ-PET and plasma ELISA are reliable diagnostic tools (Level B, Class IIa).
3. The retinal RAG gene can be utilized as a molecular biomarker for predicting visual system aging and can be clinically assessed through GWAS (Level B, Class IIa).
4. Protein alterations in tears, aqueous humors, and vitreous humors may be used as vital molecular biomarkers for predicting visual system aging, analyzed by proteomics. To firmly probe the proteins in eye fluid in the assessment of visual system aging, it is necessary to conduct subsequent cohort studies (Level B, Class I).
5. Changes in the microbiota of meibum, conjunctival sac, and eyelid skin can be regarded as molecular biomarkers for visual system aging. Utilizing 16S rRNA gene sequencing, additional clinical research is required to evaluate its practical efficacy (Level B, Class IIa).

## Clinical application of visual system aging biomarkers

Biomarkers of visual system aging hold broad clinical significance, covering early disease diagnosis, personalized treatment planning, and therapeutic efficacy assessment.

(1) Early diagnosis and screening: Identifying biomarkers of visual system aging enables the early recognition of ocular abnormalities, even before the onset of noticeable symptoms.
(2) Disease risk assessment and intervention: By tracking biomarkers of visual system aging, individual susceptibility to ocular diseases can be estimated, facilitating the development of tailored protective and therapeutic strategies.
(3) Therapeutic efficacy evaluation: The continuous monitoring of visual system aging markers is conducive to evaluating treatment effectiveness and adjusting treatment regimens in line with the patient’s aging progression.

The aging process in the visual system is a multifaceted biological process, and the exploration and application of its biomarkers are of substantial significance. By integrating functional, structural, and molecular biomarkers through comprehensive screening and analysis, robust tools can be developed to enhance ophthalmic practice, supporting early diagnosis, personalized treatment, and targeted aging intervention. As biomarker technologies continue to evolve, these advancements are anticipated to establish more precise diagnostic criteria, which will enable early detection, prevention, and management of age-related visual system disorders.

## Construction of “visual system age” prediction models

### Data collection

#### Sample selection

(1) Individuals of varying ages, genders, and living environments as study participants to ensure sample diversity and representativeness.
(2) Samples include both individuals with normal vision and those with different degrees of vision problems, such as myopia, hyperopia, and cataract.

#### Data collection

(1) A comprehensive evaluation of the visual system, including visual acuity assessment, fundus examination, and intraocular pressure measurement, among other assessments, to thoroughly evaluate visual system health.
(2) Biological samples like blood, tears, and aqueous humor were collected to monitor the levels of biomarkers related to visual system aging.
(3) Information on lifestyle, medical history, and family history as potential influencing factors.
(4) Special consideration is given to long-term eye usage habits, environmental influences (e.g. ultraviolet exposure), and a history of visual system disorders.

### Model formation

#### Selection of modeling methods

(1) Machine learning algorithms such as multivariate linear regression, support vector machines, and random forests can be employed.
(2) Nonlinear modeling methods may be more appropriate, considering visual system aging markers and the actual age.

#### Variable selection

(1) In addition to visual system aging markers, variables such as age, gender, lifestyle, ultraviolet exposure history, and visual system disease history can be integrated into the model.
(2) Employ techniques such as stepwise regression and principal component analysis for variable selection, effectively eliminating redundancy and improving the model’s accuracy and stability.

#### Model training

(1) Utilize the collected data to train the model and optimize its parameters, which enables accurate prediction of visual system age.
(2) Divide the data into a training set and test set for cross-validation to evaluate the performance of the model.

### Model evaluation and optimization

#### Evaluation indicators

(1) Commonly used evaluation indicators include mean square error, average absolute error coefficient of determination, etc. These indicators are used to measure the accuracy of model prediction.
(2) The model’s accuracy, sensitivity, and specificity can also be assessed to evaluate its predictive capability across different age groups, which ensures its effectiveness in diverse populations.

#### Model optimization

(1) Based on the evaluation results, the model can be refined by adjusting parameters, incorporating additional variables, and enhancing the modeling approach.
(2) The process of model training and evaluation was repeated until optimizing performance and prediction accuracy were achieved.

### Model application

#### Application model

The established visual system age prediction model can be applied in clinical research to provide a scientific basis for visual health assessment, disease screening, and personalized eye care strategies.

#### Validation model

(1) Additional sample data were collected to validate the model.
(2) Continuously update and improve the model as time progresses and new data accumulates to ensure its stability and generalization while minimizing prediction inaccuracies caused by data bias.

By integrating visual system aging biomarkers into an age prediction model, a more precise assessment of individual visual aging can be achieved, which enables early detection of vision issues and targeted interventions to support personalized eye health management.

## Conclusion and future perspectives

According to the expert discussion, the following 16 visual system biomarkers are recommended in the three dimensions of functional, structural, and molecular factors. These markers will be further validated in different age groups in the future (Table 3).

**Table 3.** Recommended biomarkers of visual system aging

Dimension	Biomarker	Test method	COR	LOE
Functional markers	MGD	Meibography, slit lamp examination	**IIa**	**A**
	Conjunctival folds	Slit lamp examination	**IIa**	**A**
	Tear dysfunction	TBUT, Schirmer’s test, silt lamp examination	**IIa**	**A**
	Presbyopia	Eye exam	**I**	**A**
	A-wave, B-wave, OPs	ERG	**I**	**A**
Structural markers	Corneal endothelial cell density	IVCM, OCT	**I**	**B**
	Lens opacity and elasticity, vitreous opacity	Fundoscopy, slit lamp examination	**I**	**A**
	Choroidal vasculature	OCT	**IIa**	**B**
	RAG-based fundus images	Fundus camera	**IIa**	**B**
	Retinal microvascular complexity	Fundus camera	**IIa**	**B**
	RNFL	SD-OCT	**IIa**	**C**
Molecular markers	Scleral AGEs	Raman spectroscopy	**IIb**	**C**
	Retinal Aβ	PET, ELISA	**IIa**	**B**
	RAG gene	GWAS	**IIa**	**B**
	Proteins in eye fluid	Proteomics	**I**	**B**
	Surface microbiota	16S rRNA gene sequencing	**IIa**	**B**

The working route map of visual system aging biomarker research in China delineates the following key objectives: (i) Establish a national multicenter aging cohort of about 1000 subjects to identify and validate visual system aging biomarkers, develop detection techniques and methods, determine the reference values for visual system aging biomarkers in the Chinese population, predict the “turning point” of visual system aging, and define the optimal time window for intervention. (ii) Leverage AI to establish a visual system aging assessment model and a visual system aging-related diseases prediction model, capitalizing on the unique advantages of imaging in visual system diagnostics. (iii) Advance deep collaboration of industry, academia, research, and government to propel the translation and application of scientific breakthroughs.

By integrating multidisciplinary approaches and technological innovations, we aspire to develop a more precise visual system aging assessment framework, offering a scientific foundation for the early prevention and management of age-related ocular diseases, ultimately contributing to the strategic vision of “healthy aging.”
